# 4D evolutions of cracks, voids, and corrosion products in reinforced concrete materials

**DOI:** 10.1038/s41598-023-48058-9

**Published:** 2023-12-17

**Authors:** Jaber Taheri-Shakib, Adil Al-Mayah

**Affiliations:** 1 Department of Civil and Environmental Engineering, University of Waterloo, Waterloo, ON N2L 3G1 Canada; 2 Department of Mechanical and Mechatronics Engineering, University of Waterloo, Waterloo, ON N2L 3G1 Canada

**Keywords:** Engineering, Materials science

## Abstract

This research paper presents a comprehensive investigation into the corrosion process in reinforced concrete structures using advanced analytical techniques, namely non-destructive X-ray computed tomography (CT) imaging, scanning electron microscopy (SEM) images, energy dispersive x-ray spectrometry (EDS), and Raman spectroscopy. The CT image analysis allowed for the identification and quantification of pore structures, crack propagation, and corrosion progression at different stages of corrosion. CT scanning and data analysis offer valuable 4D (3D spatial + time) insights into corrosion in reinforced concrete, revealing changes in pore sizes, with smaller pores increasing and larger pores decreasing as corrosion progresses. Our investigation reveals dynamic changes in reinforced concrete pores during the accelerated corrosion test leading to new pore formation and cracking. The research identifies two distinct types of cracks: one filled with corrosion products and the other, zipper-like cracks, resulting from the connection of deformed pores without corrosion products. The SEM images and EDS analysis confirmed the absence of corrosion products within these unique zipper cracks, suggesting a different mechanism of crack formation compared to the first type of cracks. The results revealed two distinct categories of corrosion products: iron oxides and iron hydroxides, with their distribution correlated to the duration of accelerated corrosion testing. The integration and verification of results from X-ray CT imaging and Raman spectroscopy established a comprehensive understanding of corrosion-induced damage in the reinforced concrete specimen, shedding light on complex interactions among different corrosion products during the corrosion process. These findings offer crucial insights for better understanding of the corrosion process in reinforced concrete paving the way for future development of effective treatments and strategies to mitigate corrosion impact.

## Introduction

Corrosion is the most prevalent cause of damage to reinforced concrete buildings and infrastructures resulting in significant economic losses. The process of reinforcement corrosion is a complex electrochemical phenomenon, initiated by the presence of ions, such as chloride, on the steel surface. This corrosion process leads to a decrease in the cross-sectional area of the steel bar and an increase in the volume occupied by corrosion products. This internal expansion results in tensile stresses, cracking, spalling of the concrete cover, and degradation of bond between concrete and reinforcement. Monitoring the progression of corrosion-induced damage is a challenging task using traditional inspection methods. However, understanding the mechanism of reinforcement corrosion and associated damages are of utmost importance to ensure the durability and safety of concrete structures.

Computed tomography (CT) scanning has emerged as a powerful tool for the non-destructive evaluation of concrete structures, enabling the detection, characterization, and assessment of corrosion-induced damage. CT imaging utilizes X-ray and a detector on the opposite side to record the X-ray attenuation. This attenuation data is then processed using sophisticated software algorithms to reconstruct a three-dimensional image of the object's internal structure. It produces detailed 3D images of an object or material^1^ at different time intervals of loading, environmental and chemical reactions process. In addition to its x-ray source. The advantage of CT scanning in concrete characterization is its ability to non-destructively image the internal structures of concrete including cracks, voids, and other defects that are difficult to detect using traditional inspection methods. This technique has been recently used in the civil engineering field to investigate the microstructure of concrete and to assess its durability and performance^2^. Furthermore, the images obtained from CT scans can be analyzed quantitatively to provide information about the concrete's density, porosity, additive distribution, cracks and other properties^3^.

Studies have shown the effectiveness of CT scanning in concrete characterization. For example, the CT technique has been employed to investigate the pores characteristics within concrete^4–6^. Its high-resolution enables the detection of fine cracks, micro-pores and deformations in the range of microns^7^. Overall, the CT technique is proved to be an effective tool to investigate corrosion in reinforcing steel^8^. It is worth mentioned that, detailed cross-sectional images of the internal structure of concrete using CT enable the identification of areas of localized corrosion and the determination of the depth and extent of corrosion-induced damage. Furthermore, by analyzing CT images, researchers can quantify the amount of corrosion and assess the integrity of reinforced concrete structures.

One study conducted by Ebell et al. demonstrated the effectiveness of CT scanning for the detection of corrosion in reinforced concrete under different environmental conditions^9^. The researchers were able to identify areas of localized corrosion and determine the extent of corrosion-induced damage. Qi et al.^10^ used the CT technique to specify the micro-cracks generation and propagation in reinforced concrete. Another study by Robuschi et al. evaluated the effectiveness of CT scanning to predict the consequences of corrosion damages and to quantify the amount of corrosion products and their distribution in reinforced concrete in a bridge pier that had experienced severe corrosion-induced damage^11^. In addition, CT scanning can provide detailed information about the density, porosity, and other properties of concrete, enabling researchers to assess the severity of corrosion-induced damage. This quantitative analysis can help researchers to develop effective repair and maintenance strategies for reinforced concrete structures. Van Steen et al. quantitatively analyzed the distribution of corrosion-induced damage by locating and characterizing chloride-induced corrosion damage of the bridge deck^12^. Wang et al.^13^ investigated corrosion in reinforced concrete structures exposed to a marine environment using in situ and real-time CT scanning to monitor the cracking position and quantify the amount of corrosion in the concrete.

In this research, a comprehensive analysis and assessment of the corrosion process in reinforced concrete are conducted through an accelerated galvanostatic corrosion test. To achieve this objective, the CT imaging is employed to evaluate structural changes and identify the types of corrosion products formed at various stages of the corrosion process in reinforced concrete. To further validate the CT scan results and gain deeper insights, we conduct SEM, EDS, and Raman spectroscopy tests. These complementary techniques serve to confirm the findings from the CT scan and allow us to explore and compare different proposed scenarios regarding the corrosion process. The combined use of these advanced analytical methods enables a comprehensive understanding of the corrosion mechanisms and its impact on the reinforced concrete structure, providing valuable insights for enhancing durability and maintenance strategies in real-world applications.

## Materials and methods

### Materials and specimen preparation

**Figure 1:**
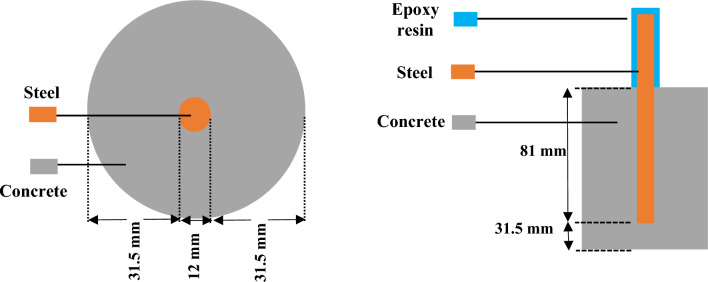
Schematic of the reinforced concrete sample.

A total of 6 concrete specimens, comprising two different sizes, were cast. These samples were investigated using the state-of-the-art 3D CT imaging during the corrosion process at various time intervals. This was followed by advanced material analysis methods namely: SEM analysis, and Raman spectroscopy at the final stage of corrosion. As presented in Fig. 1, a cylindrical specimen with a diameter of 75 mm and a height of 100 mm was prepared with a rebar of 12 mm diameter in the center. The design ensured that the thickness of the concrete cover remained consistent around the specimen, with 31.5 mm of thickness from the sides and bottom. Although, these dimensions and specifications may not represent a typical concrete structure, they were carefully considered to ensure accurate and reliable testing results. The water/cement ratio was 0.6, the sand/cement ratio was 1 and cement/aggregate ratio was 0.25. The cement used in this study was Type I Portland cement. It is typically composed of clinker, calcium sulfate, fly ash, slag, or natural pozzolans. The specimen was then sealed and placed for 24 h in a curing room at 20 ± 2  °C and under 95 ± 5% of relative humidity. After 24 h, the specimen was de-molded and returned to the curing chamber for another 28 days. Notably, epoxy coating was applied to prevent the corrosion of the exposed portion of the rebar during the curing and testing procedures^14^.

### Accelerated corrosion

**Figure 2:**
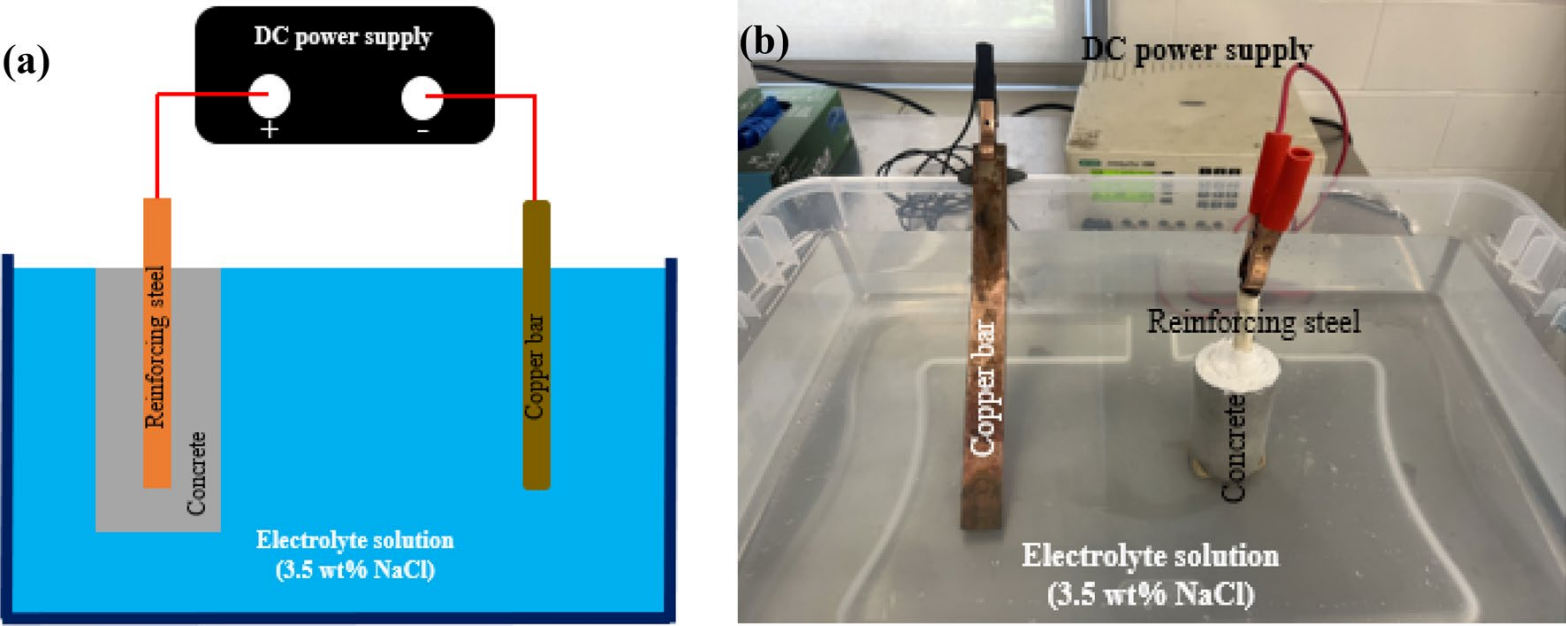
(**a**) Schematic and (**b**) experimental setup illustration of accelerated corrosion procedure.

To investigate the corrosion behavior of reinforced concrete structures, galvanostatic corrosion was applied to accelerate the process. The reinforcing steel was connected to the positive end of the DC power supply as the anode, while a copper bar was connected to the negative side as the cathode, as illustrated in Fig. 2. The concrete sample was immersed in an electrolyte solution containing 3.5 wt% NaCl. An imposed direct current with a constant density of 100 μA/cm^2^ was applied, as this represents the maximum current density observed in natural conditions according to previous studies^12, 15^. It is worth noting that for higher current densities, the internal pressure can increase rapidly as the corrosion products are unable to fill the pores within a sufficient time frame^16^. The applied current was deliberately set to 3 mA with minimal voltage during the entire process. This methodical approach serves to guarantee negligible fluctuations in the water temperature, aligning it precisely with room temperature throughout the test.

### Characterization techniques of corrosion process

#### X-ray CT imaging

The corrosion of reinforcing steel in accelerated corrosion processes was monitored using a high-resolution CT imaging system (Phoenix v tome xs 240, GE) at 72-h intervals. The obtained CT 3D images were displayed with various gray values, indicating absorption levels of different materials. Threshold division was used to distinguish reinforcing steel, corrosion products, mortar, and cracks based on their disparities in linear attenuation levels. The imaging parameters utilized were an X-ray voltage of 120 kV and a current of 70 µA, with an exposure time of 0.33 s and a voxel size of 100 µm. Each scan comprised 1015 images taken over a 360° rotation. Post-imaging processes were implemented to minimize scanning artifacts and analyze the internal structures of the reinforced concrete specimens. To analyze the collected images (i.e. segmentation, evaluation, and clear visualization), VGStudio MAX 2.2 software was utilized. These imaging parameters are deemed appropriate for capturing high-resolution CT images efficiently, and identical post-imaging processes were applied to all collected images.

#### SEM and EDS

After conducting CT imaging analysis at different time intervals during the full corrosion process, the microstructure of the mortar matrix, distribution of corrosion products, and corrosion morphology of steels within sample slices were characterized using Scanning Electron Microscopy (SEM) equipped with Energy-Dispersive X-ray Spectroscopy (EDS) (Tescan VEGA TS-5130). EDS analysis was utilized to investigate the distribution of unique elements in the mortars. To enhance conductivity, the samples were coated with a thin layer of gold using a sputter-coating technique under vacuum conditions. Subsequently, SEM analysis was performed at suitable voltage (30 kV) with a close working distance, tailored to the corrosion level of individual specimens, to improve resolution.

### Raman spectroscopy

Raman spectroscopy was employed as a powerful tool to discern the constituents of steel corrosion products. Through comparison of the obtained spectra with reference spectra from reputable literary sources, distinct phases of the corrosion product could be identified. The measurements were conducted using a Renishaw inVia Reflex confocal Raman microscope. For excitation, a 532 nm (Renishaw DPSSL laser, 50 mW) laser, filtered to 1% of its maximum intensity, was employed alongside an 1800 lines/mm diffraction grating. The Raman data obtained were then processed and analyzed using the Renishaw WiRE 5.3 software package. The spectral processing involved baseline subtraction, spectrum normalization, and curve fitting techniques to ensure accurate and reliable data interpretation.

## Results and discussion

**Figure 3:**
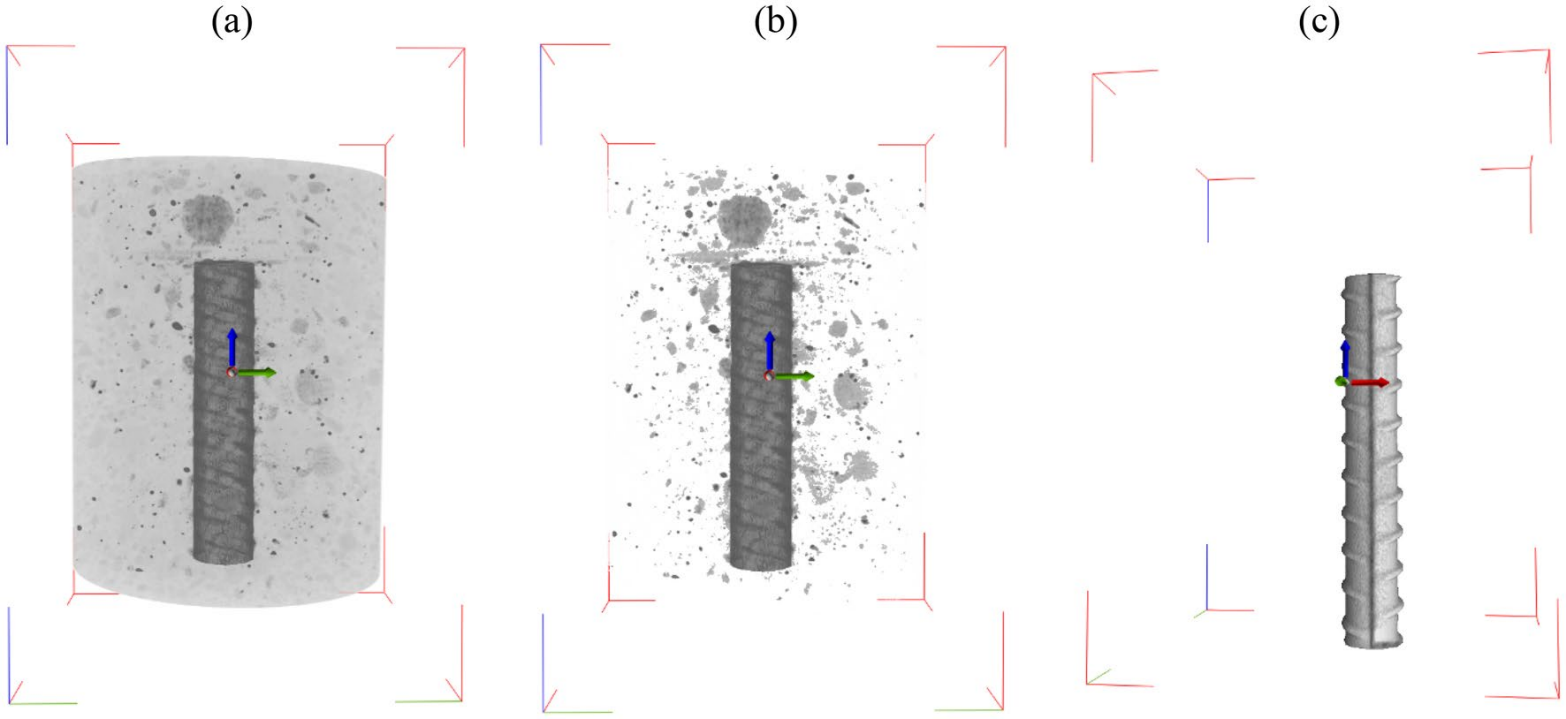
3D reconstructed tomography results of the sample. (**a**) whole sample (mortar, aggregate and steel bar) (**b**) aggregate and steel bar (**c**) steel bar.

Through comprehensive analysis and precise quantification of the reconstructed image from CT collected throughout the process of corrosion, it becomes possible to obtain distinct phases and effectively differentiate and quantify various substances. This is particularly relevant in the case of reinforced concrete, where the density of constituent materials varies, resulting in different gray levels in the CT image. As depicted in Fig. 3, the CT images of the sample, containing whole sample (mortar, aggregate and steel bar) (a), aggregate and steel bar (b), and steel bar (c), clearly distinguish the different materials. These advanced techniques enable the precise reconstruction and visualization of the internal structure of materials, providing a high level of accuracy and clarity.

**Figure 4:**
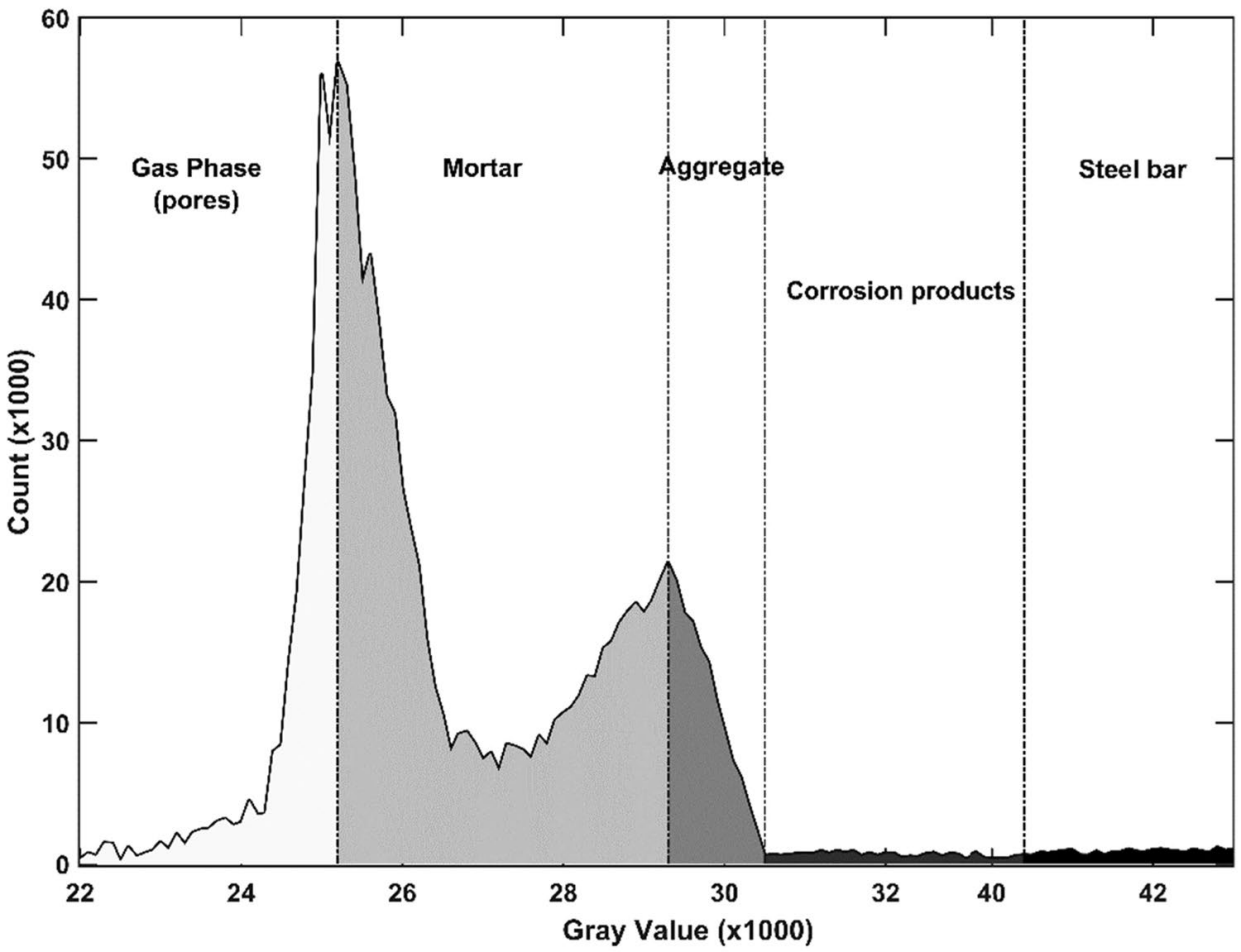
Phase segmentation of the sample image.

In this research, a technique utilizing pixel greyscale value histogram was employed to analyze and segment the multiple phases in 2D and 3D images, as shown in Fig. 4. The histogram provides valuable information regarding the distribution of pixels with specific greyscale values, enabling effective segmentation of different phases within the image. The greyscale value of each pixel is closely related to the linear attenuation coefficient, which in turn is determined by the density of the phase. Higher density results in a smaller linear attenuation coefficient and higher greyscale value. Therefore, in the case of reinforced concrete, the descending order of greyscale values corresponds to steel bars, rust, aggregates, cement matrix, and pores. To achieve a quantitative analysis of the target materials, image binarization is applied based on greyscale and morphological analysis. Binarization involves transforming the greyscale image into a binary image, where the threshold is determined by relevant information in the greyscale image corresponding to a specific grey level interval. In the resulting binary image, pixels with grey level values falling within the threshold interval are set to 1, while others are set to 0. This allows extraction of the features of the identified space from the entire dataset^17, 18^. Based on the different greyscale values, as shown in Fig. 4, the target objects including mortar, aggregate, and steel bar can be extracted, as demonstrated in Fig. 3. The precision of CT imaging in discerning substances with subtle density differences is influenced by a multitude of factors. This sophisticated approach enables precise segmentation and quantification of different phases in the reconstructed image, facilitating a high accuracy of the analysis of the materials^19^. Although, this resolution is more suitable for detecting larger structural features within the specimens, the role of these features is more significant on the fracture process of concrete than finer smaller ones. When it comes to finer distinctions in density, such as those between corrosion products, the resolution may not be sufficient to provide precise differentiation.

### Analysis of pore size distribution

**Figure 5:**
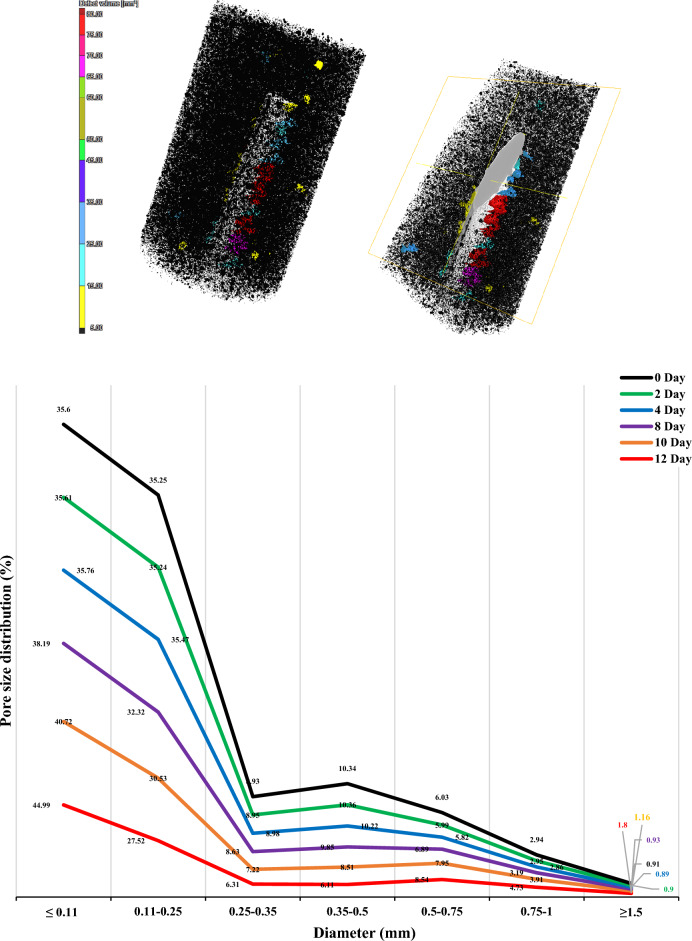
Reconstructed 3D tomography and pore distribution at different stages of accelerated corrosion tests.

The reconstruction and data analysis of CT scanning using VGStudio MAX 2.2 software provide valuable insights into the four-dimensional (4D) (i.e. 3D spatial + time) views of the process and components distribution. By employing this software, we were able to obtain a mesoscopic model that accurately reflects the structure and distribution characteristics of the pore space. Figure 5 visually presents the different size dependent color-coded distribution of pore (labeled as defect in the diagram). Additionally, Fig. 5 provides valuable information regarding the extracted pore size distribution, facilitating a quantitative description of the pore characteristics. In the study, it was observed that changes in pore size and corrosion effects stabilized after the initial 12 days of accelerated corrosion where, at this early stage, corrosion process are dynamic and critical for damage development. It is interesting to note that the proportion of small pores (≤ 0.11 mm) in concrete increases as the corrosion process progresses. In the initial sample, these small pores account for 35.6% of the total, while in the final sample (after 12 days of accelerated corrosion), they make up 44.99%. This observation suggests that these small pores are less prone to corrosion during the process^20^. This characteristic is particularly noticeable in the 4-day sample, where the ratio of these pores remains relatively constant. Also, it was been observed that pores within the size range of 0.11–0.5 mm exhibit a decrease in their proportion within the concrete as the corrosion process progresses. Specifically, pores measuring 0.11–0.25 mm, 0.25–0.35 mm, and 0.35–0.5 mm demonstrate a decreasing trend of 7.62%, 2.62%, and 4.23% respectively. On the other hand, the volumetric ratio of pores larger than 0.5 mm increases as the corrosion process continues in reinforced concrete. This enlargement of pore size is a notable characteristic of the corrosion phenomenon within the reinforced concrete structure^21^.

It is worth mentioned that the emergence of new pores in specific regions of reinforced concrete is a multifaceted phenomenon influenced by various factors inherent in the corrosion process. The electrochemical aspect plays a pivotal role, as the differential flow of electrons in areas undergoing corrosion can be attributed to variations in ion concentration, oxygen availability, and pH. Notably, the interface between aggregates and mortar serves as a focal point for pore formation, susceptible to localized corrosion when the protective passivating layer on steel reinforcement is breached. Moreover, differential mass transport, influenced by moisture content, temperature, and the presence of other chemical elements, contributes to varying rates of corrosion and pore development. The inherent heterogeneity in the microstructure of reinforced concrete, along with the influence of physical stresses, further complicates the selective formation of pores in distinct areas.

### Evaluation of pore microstructure

**Figure 6:**
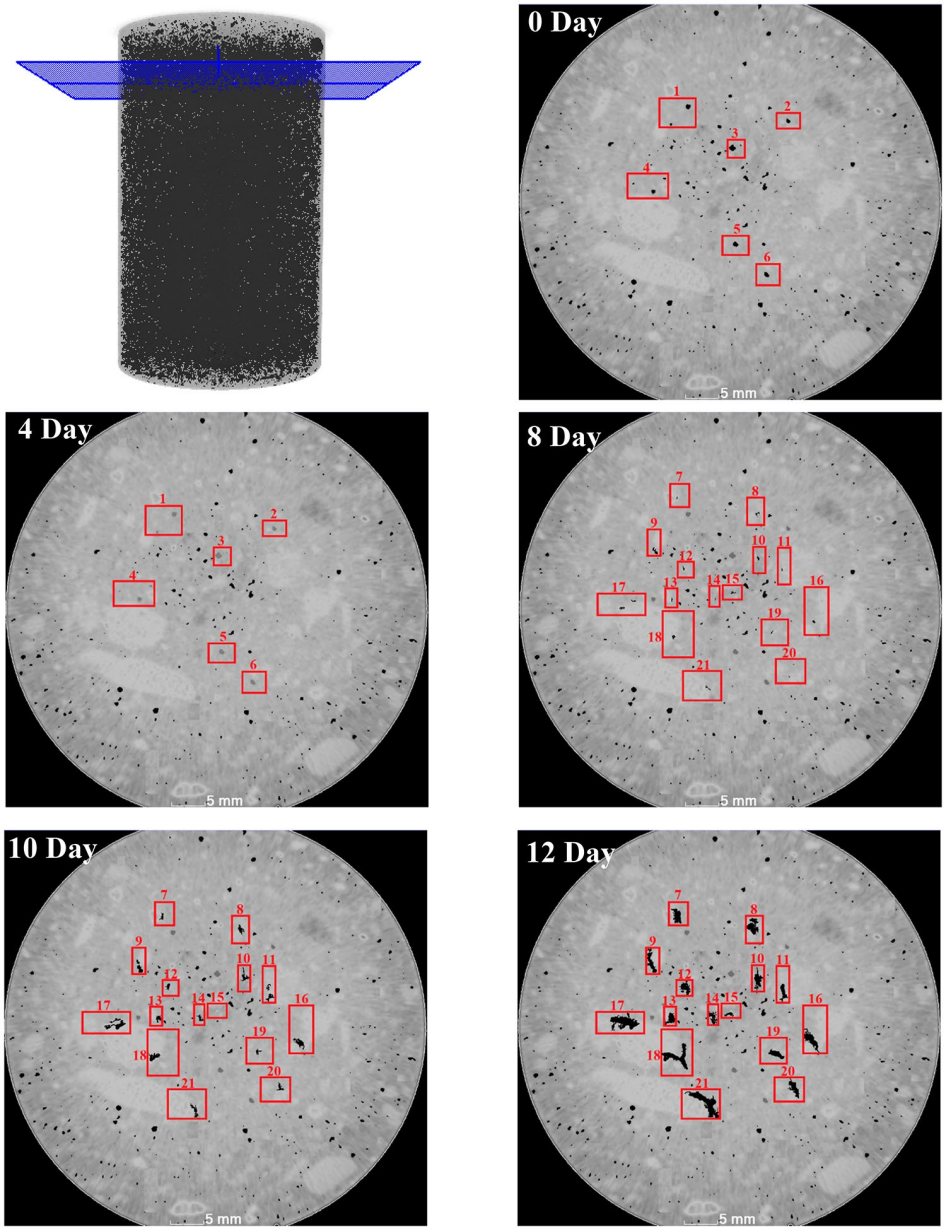
CT images depicting the pore structure in region without the reinforcing bar during various stages of accelerated corrosion.

**Figure 7:**
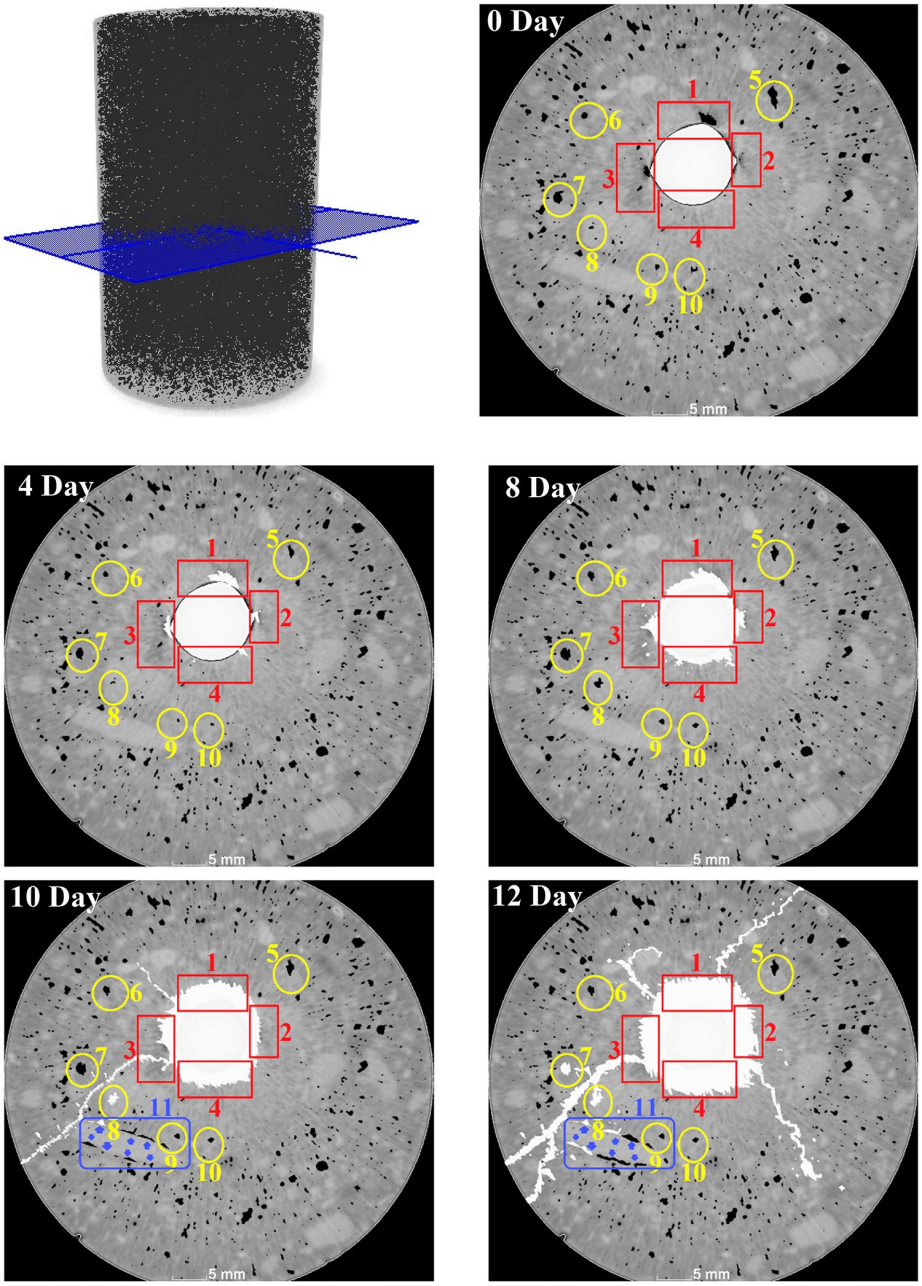
CT images of the pore structure and cracks in the vicinity of the reinforcing bar during various stages of accelerated corrosion.

To investigate the pore evolution during the corrosion cycles, the visual depiction of pores is presented in Figs. 6 and 7. In order to facilitate a comprehensive analysis, two distinct areas were examined: one without the reinforcing bar in the cross-section (Fig. 6) and another positioned halfway along the bar's height within the concrete (Fig. 7). This approach allows us to explore the pore structure in different regions of the reinforced concrete specimen. It is important to note that multiple areas were thoroughly analyzed and assessed, and the results obtained from these two specific areas are indicative and representative of the overall outcomes. In addition to pores structures, Figs. 6 and 7 illustrate the spatial distributions of aggregates, and mortar throughout various accelerated corrosion cycles. As previously mentioned, the diverse densities of concrete constituents result in varying shades of gray, enabling the differentiation of different components within the image. In these images, the aggregates are represented by the lighter gray phase, while the relatively darker phase and black regions correspond to the mortar and pores, respectively**.**

From Fig. 6, two significant changes become apparent: the filling of existing pores and the formation of new pores. After 2 days of corrosion process, certain pores/regions (labeled as 1 to 6 in the figure) no noticeable changes in pore shape and distribution were observed. However, after a 4-day period of corrosion, these pores were partially filled. Although, no new pores were formed within the 4-day timeframe, the existing pores were filled due to the migration of corrosion products. The corrosion products migration through a matrix were restricted by a fluid pressure gradient caused by the structural confinement of the bars in concrete, possibly influenced by the solubility of corrosion products^11^.

Continuing the corrosion process for 8 days reveals the formation of new pores in select areas. The newly formed pores predominantly concentrate in the central region of the reinforced concrete sample, with damage progressing from the center towards the surface. A crucial aspect of new pore formation lies in the specific areas where these pores emerge. As depicted in the figure, the majority of new pores form and grow at the interface between aggregates and mortar, encompassing most of areas 7–21, except 10, 14, 19 that were away from aggregate-mortar interface. This phenomenon holds true in other regions above the reinforcing bar, with the highest percentage of new pore formation occurring at the border areas between aggregates and mortar. Notably, in area 21, the formation of new pores is particularly prominent due to the larger size of the aggregate, resulting in increased pore formation and growth with extended durations of accelerated corrosion. This can be attributed to weak bond connection between aggregate and mortar due to weak aggregate surface. Also, in certain cases, the presence of reactive aggregates containing specific types of silica can initiate alkali-silica reaction in concrete. Alkali-silica reaction represents a chemical reaction between the alkalis present in the concrete pore solution and the reactive silica in the aggregates, leading to the generation of a gel-like substance. This gel has the capacity to absorb water and expand, ultimately resulting in the development of new pores and cracking at the interface between aggregates and mortar^22^. In other areas, we focused on analyzing the expansion and changes of pre-existing pores near the reinforcing bar. These pores showed significantly higher levels of activity and changes compared to the other mentioned areas.

### Crack propagation

The accelerated corrosion test conducted within a 4-day timeframe revealed significant observations, as depicted in Fig. 7. It is evident that the pits surrounding the reinforcing bar were filled with corrosion products, while some other pores were also compressed and consequently shrunk. This stage corresponded to an increase in pore volume in areas 1, 2, 3, and 4, primarily due to the expansion of the pits and the subsequent filling with corrosion products. Conversely, areas 5–10 displayed a decrease in pore volume, particularly noticeable in the larger pores like 5, 6, and 7. This phenomenon of increasing pit volume and its subsequent filling with corrosion products is a well-documented during corrosion process^17, 18^. The increase in volume of rust products exerts expansion forces on the surrounding concrete, resulting in mechanical stress and subsequent reduction in pore volume. This phenomenon is further exacerbated by the accumulation of corrosion products. The combination of expansion forces, induced mechanical stress within concrete, and the presence of corrosion products collectively contribute to the reduction in pore volume within the concrete affected by rebar corrosion.

Within an 8-day timeframe, significant observations can be made regarding the corrosion process. Figure 7 clearly illustrates an increase in the volume of corrosion products surrounding the reinforcing bar, ultimately covering its entire surface. Additionally, during this period, there was evidence of the migration of corrosion products towards the pores in the proximity of the reinforcing bar, particularly visible in areas 3 and 4. Furthermore, a notable alteration in the pore structure can be observed in areas 5–10 during the 8-day accelerated corrosion test. Specifically, there is an observed increase in the volume of these pores. These findings shed light on the dynamic nature of the corrosion process within the given timeframe. The increase in the volume of corrosion products surrounding the reinforcing bar signifies the progression of corrosion. The migration of these products towards nearby pores indicates their infiltration into the concrete matrix. Moreover, the observed changes in the pore structures highlight the evolving nature of the corrosion-induced damage.

As the accelerated corrosion process reached to the 10-day mark, several noteworthy developments were observed. Firstly, there was a noticeable increase in the volume of corrosion products, accompanied by the formation of cracks. It became apparent that the corrosion products were crept into these newly formed cracks, indicating a direct relationship between crack growth and the presence of corrosion products^23^. Moreover, certain pores situated along the growth path of a prominent crack on the outer surface of the sample exhibit two distinct phenomena. These pores were either interconnected by the crack on the surface or were filled with corrosion products. This phenomenon can be attributed to the accumulation of corrosion products, which leads to the development of radial pressure and hoop stress in close proximity to the reinforced bar. Consequently, when the tensile stress in the circumferential direction exceeds the tensile strength of the concrete matrix, cracking commences, marking the initiation of the cracking stage. During this phase, corrosion-induced cracks propagate within the concrete matrix, accompanied by the gradual diffusion of corrosion rust into both the concrete matrix itself and pre-existing cracks^24, 25^. Another noteworthy observation pertains to the formation of new pores with a crack-like structure at the interface between the aggregate and mortar, as depicted in area 11. At this stage, some of these newly formed pores also were filled with corrosion products. It is important to highlight that corrosion-induced cracks predominantly emerged along the interfacial transitional regions between the aggregate and cement matrix, rather than occurring preferentially within internal defects such as pores. This is because the interfacial transitional regions represent the weakest areas, susceptible to crack initiation under the expansive pressure induced by corrosion^19^.

After 12 days of corrosion, the anticipated increase in the volume of corrosion products was observed. The long crack that formed in the previous stage (from area 3 in Figure &) and extended to the outer surface of the concrete sample exhibits further development in this stage, with its opening expanding and an additional branch being formed. Moreover, the pores surrounding this crack were more exposed to corrosion products, as evident in areas 7 and 8. It is worth noting that these pores were connected to the crack, establishing a direct pathway for the ingress of corrosion products. Area 11 experienced an increase and development in the volume of pores located on the surface between the aggregate and mortar. Consequently, there was a greater penetration and deposition of corrosion products within these pores. Interestingly, the growth pattern of the fluff observed between areas 1 and 3, which had reached almost the middle of the sample, undergoes a change in direction, branching out. This change in direction aligns with the formation of pores at the interface between the aggregate and mortar, further indicating the influence of pore formation in this region on crack growth and propagation. Notably, a crack started from zone 1 and extended towards the outer surface of the concrete, while another crack emerged and progressed from the area between 2 and 4. It is important to highlight the distinctive characteristic of this second crack, as it exhibited a zipper-like growth pattern. This implies that the crack was created and expanded due to the connection between existing pores. The phenomenon of crack growth resulting from pore connection in the concrete matrix during the corrosion process has been observed in previous studies as well^26–28^. However, it should be noted that the appearance of zipper cracks in this sample occurred subsequent to cracks with different growth patterns.

**Figure 8:**
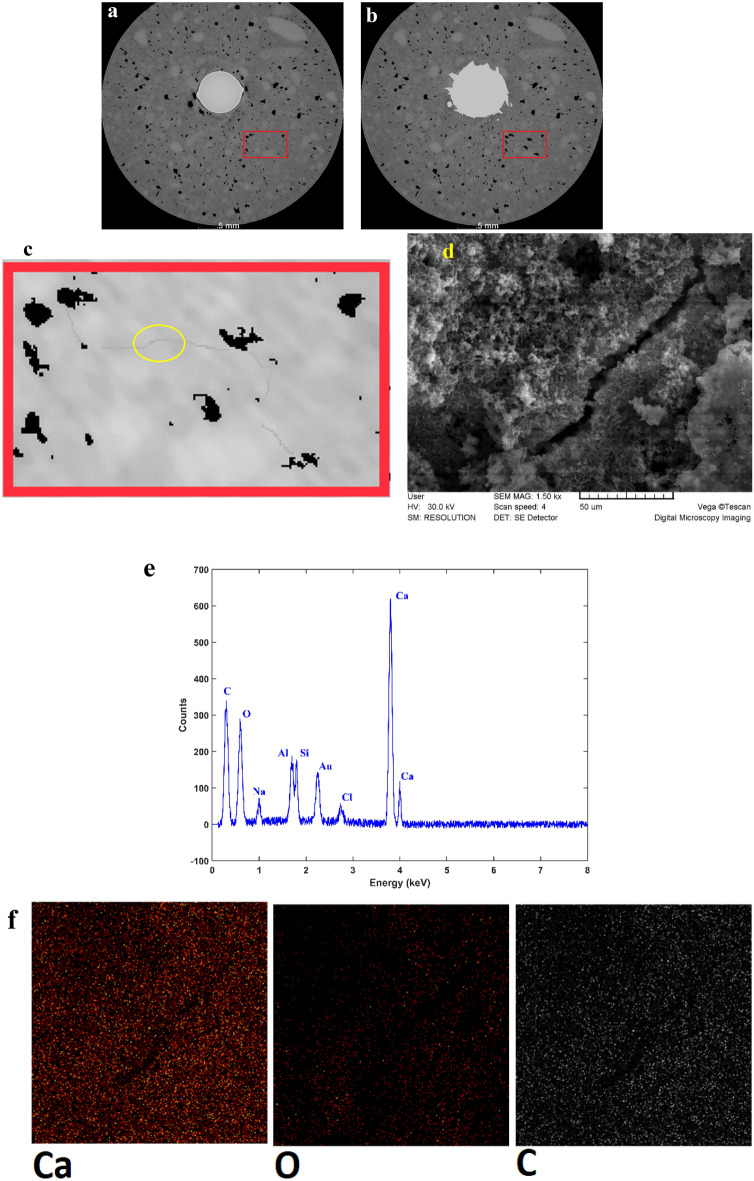
(**a**) CT scan image of the prototype. (**b**) CT scan image of the sample after 8 days of accelerated corrosion test. (**c**) Zipper microcrack development in CT scan images. (**d**) SEM image of the micro-crack formed. (**e**) EDS analysis of the specified section. (**f**) Elemental mapping of the selected section.

A close look at the corrosion process in reinforced concrete provides a clear observation that there are two main types of cracks. The first type is primarily filled with corrosion products, a phenomenon well-documented by researchers in this field^19, 29, 30^. These products are deposited within these cracks, as discussed earlier. However, another group of cracks emerges during the corrosion process in reinforced concrete, and these are not filled with corrosion products. These cracks typically exhibit a zipper-like behavior and usually have no direct connection to the reinforcing bar or the surrounding pits during their formation. Instead, they are created due to the connection of pores in the concrete matrix. An example of such zipper cracks is presented in Fig. 8. On the 10th day of the accelerated corrosion test, these cracks were observed. As the corrosion process continued, these cracks were developed, and although their openings remained relatively constant, they progressed further through the connection, of the deformed pores under the influence of the corrosion of the reinforcing rod, as previously seen in Fig. 7. Similar zipper cracks were observed in other areas as well. In fact, these cracks appeared after a significant volume of corrosion products was formed in the concrete matrix. In Fig. 8a,b, the marked area displayed an increase in the volume of existing pores, and a thin crack was formed between them, connecting them to each other. To enhance the clarity of the crack, we increased the contrast of the image (Fig. 8c). To corroborate and analyze the results of the CT scan, SEM images and EDS analysis were taken from this area (Fig. 8d,e). Notably, the EDS analysis of this zipper microcrack indicates the absence of corrosion products, consistent with the CT scan results. The mapping taken from this area further validatesd these findings (Fig. 8f). It is important to acknowledge that, in some zipper cracks shown earlier, corrosion products were found to fill them (Fig. 7). These observations provide crucial insights into the complex behavior of cracks during the corrosion process in reinforced concrete. The presence of zipper cracks, and their connection to pore deformation rather than corrosion product deposition, highlight the multifaceted nature of corrosion-induced damage in concrete structures.

### Corrosion products development

**Figure 9:**
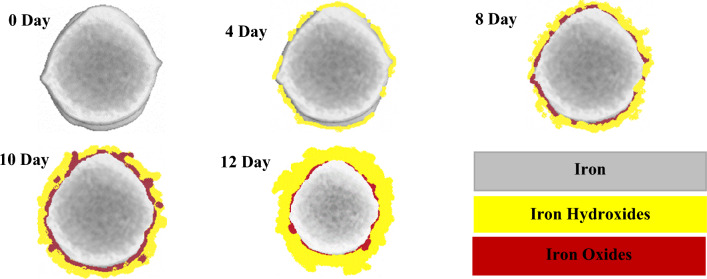
The reconstructed tomography of corrosion products during galvanostatic Tests.

Based on the X-ray CT image analysis mentioned earlier, the corrosion products present within the specimen can be identified and quantified. To achieve a clear identification of different types of corrosion products, more fundamental information must be collected, including the linear attenuation coefficients of feasible corrosion products^31–33^ and the CT number of these products^34, 35^. Building on the findings of Fang et al.^18^ products belonging to the same category exhibited closely related linear attenuation coefficients. For instance, iron oxides (FeO, Fe_2_O_3_, and Fe_3_O_4_) fall under one category, while the different categories of corrosion products can be distinguished effortlessly. The iron oxide category exhibits the highest linear attenuation coefficients, followed by the category of iron hydroxides (Fe(OH)_2_ and Fe(OH)_3_). To determine the corresponding greyscale values, the CT numbers can be calculated using an interpolation method. As a result, areas containing iron oxides (FeO, Fe_2_O_3_, and Fe_3_O_4_) exhibited the highest greyscale intensity, while those with iron hydroxides (Fe(OH)_2_ and Fe(OH)_3_) displayed a slightly lower greyscale intensity. As illustrated in Fig. 9, the CT scan images revealed two distinct categories of corrosion products with different colors. Primary iron was identified in gray, while iron oxides, including Fe_2_O_3_ and Fe_3_O_4_, appeared in red, and iron hydroxides, such as (Fe(OH)_2_ and Fe(OH)_3_), were identified in yellow. Initially, the steel reinforcing bar exhibited no products on its surface. However, after a 4-day accelerated corrosion test, corrosion products became apparent, specifically iron hydroxides around the reinforcing bar. Iron oxides were not observed at this stage. As the test progressed to the 8-day stage, iron oxides were also detected. These iron oxides were situated closer to the steel surface and exhibited a smaller volume compared to the iron hydroxides. Over the course of 10 days, a higher volume of iron oxides formed, nearly enveloping the entire surface of the reinforcing rod. At this stage, interactions between different types of corrosion products were evident. As the corrosion process continued, it became noticeable that the volume of iron oxides was significantly less compared to iron hydroxides (12 days). This is mainly attributed to the conversion of iron oxides to iron hydroxides, which was also reported by^19^. After the 12-day galvanostatic test, we observed a notable interconversion and mixing of different types of corrosion products, indicating complex interactions among the corrosion products within the specimen. Throughout all the corrosion stages where iron hydroxides were present, they consistently appeared closer to the surface of the steel bar than iron oxides.

**Figure 10:**
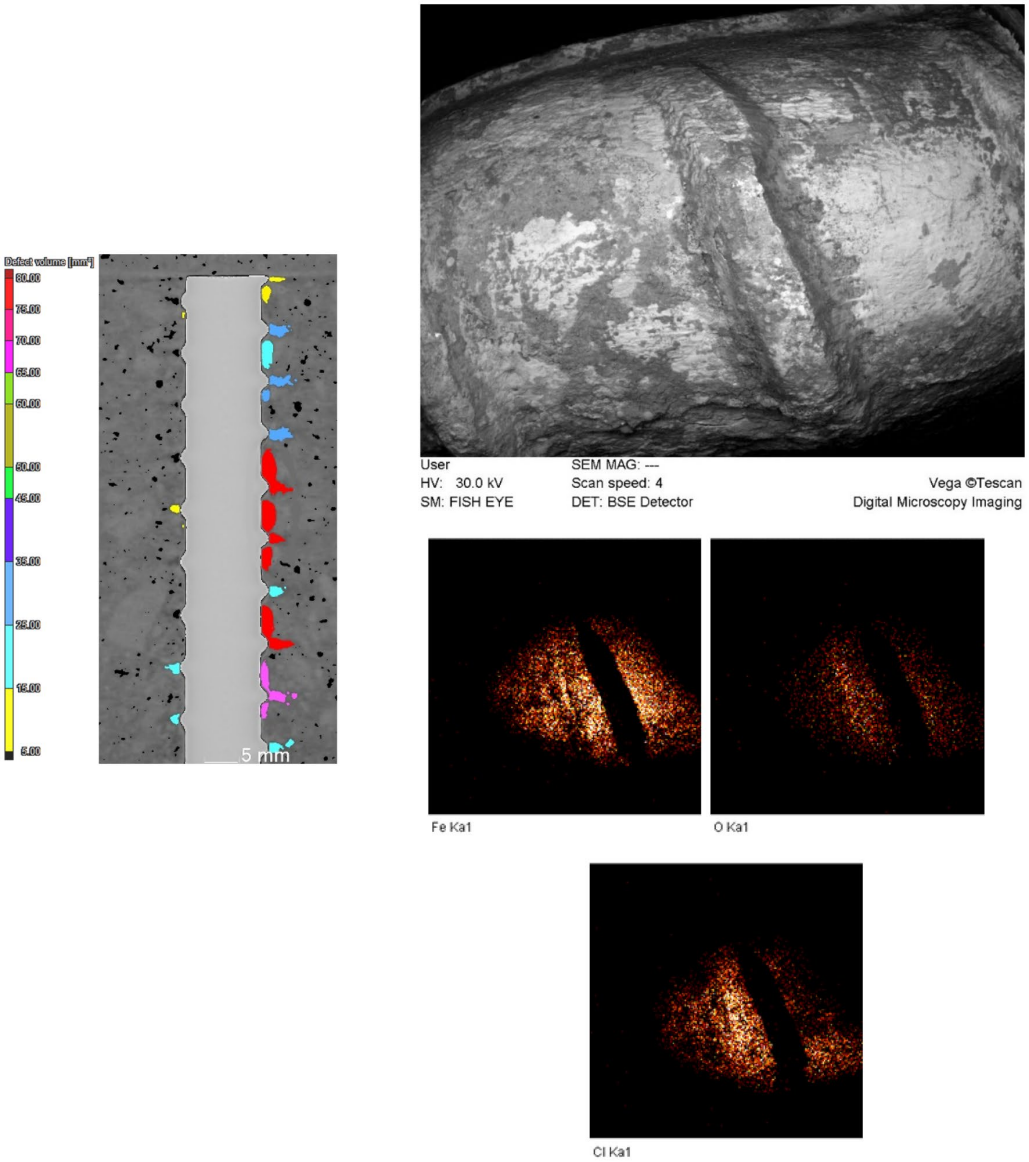
The reconstructed tomography of the initial reinforced concrete and SEM images, along with mapping, taken from the marked zone after the 12-day accelerated corrosion test.

The corrosion process and production are initiated at the largest pits surrounding the reinforcing bar (Red area in Fig. 10). The CT data analysis revealed that these pits, which covered a significant area of the rod's surface, contain the largest volume of corrosion products. Notably, the corrosion products predominantly form within a localized region characterized by the maximum contact between the rod and the pit, leading to deep penetration into the steel body and facilitating rapid corrosion. The high susceptibility of steel in these areas is attributed to the structural heterogeneity of the mortar and the presence of a porous layer at the steel/mortar interface. The mortar heterogeneities influenced the penetration pattern of aggressive ions, such as chloride ions, while the porous layer regulated the corrosion environment near the steel surface. The SEM images from this area clearly displayed the morphology and surface damage of the reinforcing rod, further confirming the occurrence of corrosion. Additionally, the mapping analysis corroborated the high levels of oxygen, indicating severe corrosion, and the presence of chloride ions on the rod's surface in this region.

In order to determine the composition of corrosion products at the interface, we conducted Raman micro-spectroscopy analyses. Most phases exhibited characteristic peaks in their spectra, enabling their identification (Table 1). However, there are four phases—magnetite, maghemite, feroxyhyte (δ-FeOOH), and ferrihydrite—that shared a common band in the 700 cm^−1^ region, making it challenging to differentiate in mixed samples within the analyzed volume. Ferrihydrite and feroxyhyte in their 2 and 6 line forms were particularly difficult to distinguish using Raman spectroscopy, as their spectra exhibit a broad band spanning from 540 to 810 cm^−1^ ^36, 37^. Maghemite also presents a wide band ranging from 600 to 800  cm^−1^, with two visible shoulders at 668 and 721  cm^−1^, the latter being more intense. Additionally, secondary weaker peaks appeared on the maghemite reference spectrum at 192, 341, 381, 458, 479, and 511  cm^−1^. On the other hand, magnetite displays a distinct pattern, featuring a strong peak at 666  cm^−1^. Despite these challenges, the differences observed in the spectra, even in mixed samples, still allowed us to detect the presence of magnetite and maghemite and effectively distinguished these two phases from each other^38^.

**Table 1:** Peak position of the phases present in corrosion products in reinforced concrete^38^.

Phase	Peak and shoulders observed on the reference spectra (cm^−1^)
Goethite (α-FeOOH)	203, 244, 300, 387, 399s, 415s, 480, 552, 684, 1002, 1113, 1304
Akaganéite (β-FeOOH)	139, 308, 331s, 389, 420s, 499, 539, 609, 720, 1410
Lepidocrocite (γ-FeOOH)	166, 217, 251, 310, 350, 378, 529, 655, 713, 1300
Hematite (α-Fe_3_O_4_)	228, 250, 294, 414, 502, 625, 670, 1330
Magnetite (Fe_3_O_4_)	306, 538, 666
Maghemite (γ-Fe_2_O_3_)	339–386, 461–512, 671–717, 1430
Wüstite (FeO)	471, 653

**Figure 11:**
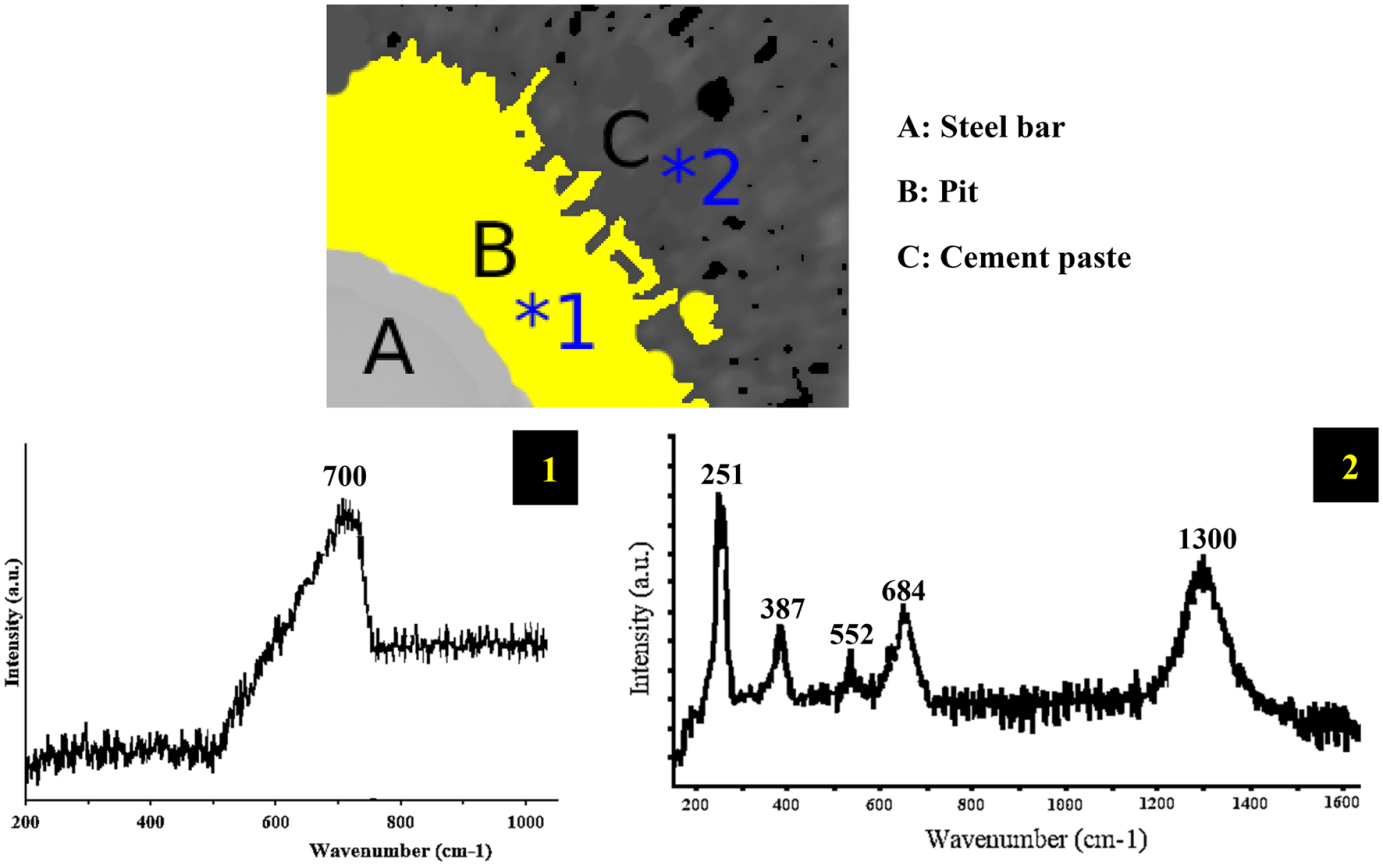
Raman spectra analysis of corrosion products in reinforced concrete following a 12-day accelerated corrosion test.

Figure 11 presents the Raman spectroscopy results obtained from the designated region. The marked area indicates an absence of cracks and underwent an accelerated corrosion test for 12 days. It is crucial to emphasize that not all structures of iron oxides provide the same level of protection^39^. Certain oxides, such as lepidocrocite (γ-FeOOH), akaganeite (β-FeOOH), iron hydroxide (FeOOH), and ferroxyhite (δ-FeOOH), have been found to be unstable and non-protective^40, 41^. In contrast, other oxide phases, including maghemite (γ-Fe_2_O_3_) and goethite (α-FeOOH), exhibit higher stability and possess a more compact morphology^42^.

Based on the Raman spectroscopy results presented in Fig. 11, the pits surrounding the reinforcement rod (region B) exhibited the presence of less crystalline phases, specifically ferrihydrite type (5Fe_2_O_3_, 9H_2_O)^43–45^. Notably, all four phases of magnetite, maghemite, ferroxyhyte (δ-FeOOH), and ferrihydrite were observed in the 700 cm^−1^ band, which aligns perfectly with the CT scan findings discussed earlier, confirming the presence of iron oxides and iron hydroxides in this area. On the other hand, area C, which related to the concrete matrix near the pits surrounding the reinforcing bar, revealed different results from Raman spectroscopy. Specifically, the presence of Lepidocrocite is detected in the bands of 251 and 1300 cm^−1^, while Goethite is identified in the bands of 387, 552, and 684 cm^−1^. Remarkably, the absence of iron oxides (Hematite, Magnetite, Maghemite, and Wüstite) in this region strongly suggests that the corrosion process leads to the transformation of iron oxides into iron hydroxide, which subsequently penetrates into the concrete matrix.

As previously discussed in the CT scan results (Fig. 9), the quantities of iron oxides decreased with the progression of the accelerated corrosion process, and correspondingly, the levels of iron hydroxides increase. This is mainly attributed to the transformation of iron oxides (Fe_2_O_3_ or rust) to iron (III) hydroxide (Fe(OH)_3_) in the presence of water which continues over time. The presence of larger volume porous structure of iron hydroxides imposes mechanical stress on the surrounding concrete promoting pore formation and cracking, ultimately contributing to concrete deterioration. The specific mechanisms may vary depending on environmental conditions, the presence of aggressive ions such as chlorides, and the concrete's unique characteristics^46–48^.

This transformation is significant since iron oxides like hematite are recognized as unstable and non-protective corrosion products for steel reinforcements in existing literature^39, 49^. However, the presence of less crystalline phases, such as ferroxyite (δ-FeOOH) and ferrihydrite (5Fe_2_O_3_·9H_2_O), suggests the potential transformation of these amorphous iron oxyhydroxides into the more stable goethite (α-FeOOH). Indeed, the CT scan results further supported our findings, as they revealed that iron oxides were predominantly absorbed onto the surface of the reinforcing rod. Subsequently, the surrounding environment was occupied by iron hydroxides, which is in complete agreement with our observations from Raman spectroscopy. As the corrosion process continued, the transformation of iron oxides into iron hydroxides became evident, and these hydroxides tended to penetrate into the concrete matrix, resulting in an increase in volume. This consistent pattern reinforces the significance of iron oxide-to-hydroxide transformation during the corrosion process, and highlights its potential implications for the overall integrity and durability of the reinforced concrete structure.

**Figure 12:**
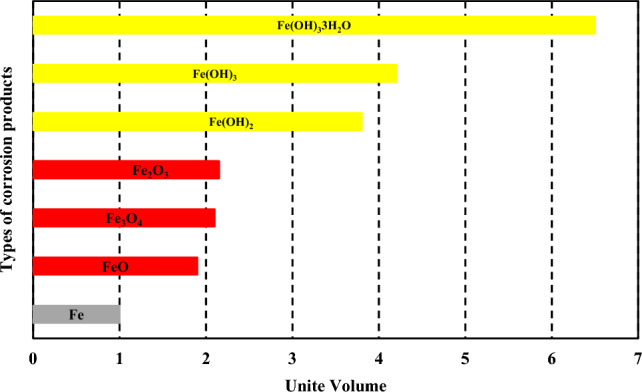
Quantification of metallic iron and its corrosion products by relative volumes^18, 50, 51^.

The formation of corrosion products typically results in volumetric expansions in the original steel. Figure 12 illustrates varying levels of volumetric expansion of corrosion products, assuming no pores exist within the products^50, 51^. It is worth noting that the presence of pores within the corrosion products would further increase their volume^18^. From both Fig. 9 and Fig. 12, we observe that when iron hydroxides are the dominating elements among the corrosion products, the volumetric expansion ratio reaches its highest level. This can be attributed to iron hydroxides having the highest relative volume compared to metallic iron. Moreover, the volumetric expansion of corrosion products is a dynamic process, influenced by the composition and properties of the corrosion products^52^. The process of transformation between metallic iron and various types of corrosion products created a recycling interaction. This interaction played a role in the evolution of corrosion products in response to changes in the surrounding environment. Consequently, the interaction between different types of corrosion products leads to dynamic changes in their relative percentages over time (as depicted in Fig. 9). The occurrence of zipper cracks during the 10-day stage of the accelerated corrosion test can be attributed to the conversion of iron oxides to iron hydroxides, which results in a significant increase in volume and subsequently exerts higher pressure. As the transformation of corrosion products commences and reaches a certain threshold, zipper cracks start to form. This indicates a substantial increase in the volume of corrosion products, leading to elevated pressure on the concrete matrix. This phenomenon holds great significance in the development of main cracks throughout the corrosion process. Understanding the factors influencing zipper crack formation and its correlation with the transformation of corrosion products is crucial for comprehending the overall behavior and durability of reinforced concrete structures experiencing corrosion threat.

## Conclusion

The present study focused on investigating the pore size distribution and evolution, formation of corrosion-induced cracks, and distribution of corrosion products during the corrosion process of reinforced concrete. The investigation covered both the steel–concrete interface and cement paste regions. Based on the findings, the following concluding remarks can be made:

- The CT scanning reconstruction and data analysis provide valuable insights into the three-dimensional aspects of the corrosion process in reinforced concrete. The obtained mesoscopic model accurately depicts the structure and distribution of the pore space, while the extracted pore size distribution facilitates a quantitative understanding of pore characteristics. The research reveals that volumetric percentage of small pores (≤ 0.11) in concrete increase as the corrosion process progresses, suggesting their relative resistance to corrosion. Conversely, pores within the size range of 0.11–0.5 mm demonstrate a decreasing trend, while larger pores (> 0.5 mm) show an increase, all of which contribute to the notable characteristics of corrosion in reinforced concrete.
- Our investigation of pore evolution during the accelerated corrosion test cycle sheds light on the dynamic changes in reinforced concrete pores. The visual depictions of different areas away from and within the reinforcing bar vicinity provided valuable insights into the distribution and formation of pores during the corrosion process. Notably, for example, the presence of reactive aggregates played a significant role in initiating alkali-silica reaction, leading to the development of new pores and cracking.
- The observations at different timeframes reveal significant changes in pore volume, migration of corrosion products, and the initiation and propagation of cracks. The infiltration of corrosion products into pores and their influence on crack growth indicate a direct relationship between pore evolution and corrosion-induced damage. Additionally, the emergence of zipper-like cracks highlights the complexity of crack formation within the concrete matrix during the corrosion process.
- The study has revealed two distinct types of cracks. The first type is filled with corrosion products, as extensively reported by previous researchers. In contrast, a second group of cracks, referred to as zipper cracks, emerges during the corrosion process, and these cracks are not filled with corrosion products. Instead, they result from the connection of deformed pores within the concrete matrix. These zipper cracks exhibit a unique behavior and are not directly linked to the reinforcing bar or surrounding pits. Also, zipper cracks appear after a substantial volume of corrosion products has formed in the concrete matrix, suggesting a different mechanism of crack formation compared to the first type of cracks. SEM images and EDS analysis further support the absence of corrosion products within these cracks.
- The differentiation of corrosion products based on their linear attenuation coefficients and CT numbers has allowed us to distinguish between iron oxides and iron hydroxides, providing clear visualization of their distribution. The analysis of the largest pits surrounding the reinforcing bar has revealed that they contain the largest volume of corrosion products. The structural heterogeneity of the mortar and the presence of a porous layer at the steel/mortar interface contribute to the high susceptibility of steel in these areas, facilitating rapid corrosion. Based on results of Raman spectroscopy, the transformation of iron oxides into iron hydroxides during the corrosion process has been observed, with less crystalline phases detected in the early stages, gradually transforming into more stable goethite. Overall, the verification of results obtained from X-ray CT image analysis and Raman spectroscopy enhances the credibility of the research findings. The combined approach of these analytical techniques provides comprehensive and detailed information about the distribution and composition of corrosion products during the corrosion process in reinforced concrete.

## Data Availability

The datasets used and/or analysed during the current study available from the corresponding author on reasonable request.
